# Supplementary material for: Coherent-State-Based Twin-Field Quantum Key Distribution
Source: Sci Rep. 2019 Oct 17;9:14918. doi: 10.1038/s41598-019-50429-0 (PMC6797752; doi:10.1038/s41598-019-50429-0)
Supplement: Supplementary file 1 — Supplemental Material [file 41598_2019_50429_MOESM1_ESM.pdf]

Supplemental Material for "Coherent-state-based twin-field quantum key distribution"

Hua-Lei Yin[1,*] and Zeng-Bing Chen[1,†]

[1]National Laboratory of Solid State Microstructures and School of Physics, Nanjing University, Nanjing 210093, China

I. HERALDED ENTANGLEMENT GENERATION

The four two-mode entangled coherent states (ECSs) [1] can be written in different forms

$$\begin{aligned}
\left|\Phi^{\pm}\right\rangle &= \frac{1}{\sqrt{N_{\pm}}}\left(|\alpha\rangle\,|\alpha\rangle \pm |-\alpha\rangle\,|-\alpha\rangle\right) = \frac{1}{\sqrt{N_{\pm}}}\left(\left|\xi^{+}(\alpha)\right\rangle\left|\xi^{\pm}(\alpha)\right\rangle + \left|\xi^{-}(\alpha)\right\rangle\left|\xi^{\mp}(\alpha)\right\rangle\right)\\
&= \frac{1}{\sqrt{N_{\pm}}}\left(\left|\xi^{+i}(\alpha)\right\rangle\left|\xi^{\mp i}(\alpha)\right\rangle + \left|\xi^{-i}(\alpha)\right\rangle\left|\xi^{\pm i}(\alpha)\right\rangle\right),\\
\left|\Psi^{\pm}\right\rangle &= \frac{1}{\sqrt{N_{\pm}}}\left(|\alpha\rangle\,|-\alpha\rangle \pm |-\alpha\rangle\,|\alpha\rangle\right) = \frac{1}{\sqrt{N_{\pm}}}\left(\left|\xi^{\pm}(\alpha)\right\rangle\left|\xi^{+}(\alpha)\right\rangle - \left|\xi^{\mp}(\alpha)\right\rangle\left|\xi^{-}(\alpha)\right\rangle\right)\\
&= \frac{1}{i\sqrt{N_{\pm}}}\left(\left|\xi^{\pm i}(\alpha)\right\rangle\left|\xi^{+i}(\alpha)\right\rangle - \left|\xi^{\mp i}(\alpha)\right\rangle\left|\xi^{-i}(\alpha)\right\rangle\right),
\end{aligned} \tag{1}$$

where the parameters $N_{\pm} = 2(1 \pm e^{-4\mu})$ are the normalization factors. The quantum states $|\pm\alpha\rangle$ are the coherent states containing $\mu = |\alpha|^2$ photons on average. The quantum states $\left|\xi^{\pm}(\alpha)\right\rangle = (|\alpha\rangle \pm |-\alpha\rangle)/\sqrt{2}$ and $\left|\xi^{\pm i}(\alpha)\right\rangle = (|\alpha\rangle \pm i\,|-\alpha\rangle)/\sqrt{2}$ are the non-normalized single-mode cat states. Considering a lossless and symmetric beam splitter (BS), the evolution of four ECSs after passing through the BS can be given by

$$\begin{aligned}
\left|\Phi^{+}\right\rangle_{ab} &\xrightarrow{\text{BS}} \frac{2e^{-\mu}}{\sqrt{N_{+}}}\sum_{n=0}^{\infty}\frac{(\sqrt{2}\alpha)^{2n}}{\sqrt{(2n)!}}\,|2n\rangle_{\tilde{a}}\,|0\rangle_{\tilde{b}} \Longrightarrow |\text{even}\rangle_{\tilde{a}}\,|0\rangle_{\tilde{b}},\\
\left|\Phi^{-}\right\rangle_{ab} &\xrightarrow{\text{BS}} \frac{2e^{-\mu}}{\sqrt{N_{-}}}\sum_{n=0}^{\infty}\frac{(\sqrt{2}\alpha)^{2n+1}}{\sqrt{(2n+1)!}}\,|2n+1\rangle_{\tilde{a}}\,|0\rangle_{\tilde{b}} \Longrightarrow |\text{odd}\rangle_{\tilde{a}}\,|0\rangle_{\tilde{b}},\\
\left|\Psi^{+}\right\rangle_{ab} &\xrightarrow{\text{BS}} \frac{2e^{-\mu}}{\sqrt{N_{+}}}\sum_{n=0}^{\infty}\frac{(\sqrt{2}\alpha)^{2n}}{\sqrt{(2n)!}}\,|0\rangle_{\tilde{a}}\,|2n\rangle_{\tilde{b}} \Longrightarrow |0\rangle_{\tilde{a}}\,|\text{even}\rangle_{\tilde{b}},\\
\left|\Psi^{-}\right\rangle_{ab} &\xrightarrow{\text{BS}} \frac{2e^{-\mu}}{\sqrt{N_{-}}}\sum_{n=0}^{\infty}\frac{(\sqrt{2}\alpha)^{2n+1}}{\sqrt{(2n+1)!}}\,|0\rangle_{\tilde{a}}\,|2n+1\rangle_{\tilde{b}} \Longrightarrow |0\rangle_{\tilde{a}}\,|\text{odd}\rangle_{\tilde{b}}.
\end{aligned} \tag{2}$$

In the virtual entanglement-based protocol, the entangled state prepared by Alice can be written as

$$\begin{aligned}
|\psi\rangle_{a'a} &= \frac{1}{\sqrt{2}}\left(|+z\rangle_{a'}\,|\alpha\rangle_{a} + |-z\rangle_{a'}\,|-\alpha\rangle_{a}\right)\\
&= \frac{1}{\sqrt{2}}\left(|+x\rangle_{a'}\left|\xi^{+}(\alpha)\right\rangle_{a} + |-x\rangle_{a'}\left|\xi^{-}(\alpha)\right\rangle_{a}\right)\\
&= \frac{1}{\sqrt{2}}\left(|+y\rangle_{a'}\left|\xi^{-i}(\alpha)\right\rangle_{a} + |-y\rangle_{a'}\left|\xi^{+i}(\alpha)\right\rangle_{a}\right),
\end{aligned} \tag{3}$$

*Electronic address: hlyin@nju.edu.cn

†Electronic address: zbchen@nju.edu.cn

and the entangled state prepared by Bob can be written as

$$\begin{aligned} |\psi\rangle_{b'b} &= \frac{1}{\sqrt{2}}\left(|{+z}\rangle_{b'}|\alpha\rangle_b + |{-z}\rangle_{b'}|{-\alpha}\rangle_b\right) \\ &= \frac{1}{\sqrt{2}}\left(|{+x}\rangle_{b'}|\xi^{+}(\alpha)\rangle_b + |{-x}\rangle_{b'}|\xi^{-}(\alpha)\rangle_b\right) \\ &= \frac{1}{\sqrt{2}}\left(|{+y}\rangle_{b'}|\xi^{-i}(\alpha)\rangle_b + |{-y}\rangle_{b'}|\xi^{+i}(\alpha)\rangle_b\right), \end{aligned} \tag{4}$$

where qubit states $|\pm z\rangle$, $|\pm x\rangle$ and $|\pm y\rangle$ are the eigenstates of Pauli's Z, X and Y operators. The bipartite qubit entanglement states $\rho_{a'b'}$ between Alice and Bob are generated by using the event-ready detection to implement upon the flying optical pulses, called entanglement swapping. Once Alice and Bob share qubit entanglement states $\rho_{a'b'}$ even with noise, they can exploit most previous security proof techniques to obtain secret key. Here, we use the entanglement purification techniques [2–4] to prove the security of our protocols against coherent attacks in the asymptotic regime.

II. ENTANGLEMENT PURIFICATION AND SECURITY PROOF OF QUANTUM KEY DISTRIBUTION

Here we review the entanglement distillation protocol (EDP) of bipartite qubit systems and its relation with the security proof of quantum key distribution (QKD). In the work of Bennett, Divincenzo, smolin and Wooters (BDSW) [5], it was shown that any bipartite qubit system density matrix can always be transformed into a diagonal form by local operations and classical communication. The diagonal forms of density matrix are in the Bell states:

$$\begin{aligned} |\psi_1\rangle &= \frac{1}{\sqrt{2}}(|{+z}\rangle|{+z}\rangle + |{-z}\rangle|{-z}\rangle), \\ |\psi_2\rangle &= \frac{1}{\sqrt{2}}(|{+z}\rangle|{+z}\rangle - |{-z}\rangle|{-z}\rangle), \\ |\psi_3\rangle &= \frac{1}{\sqrt{2}}(|{+z}\rangle|{-z}\rangle + |{-z}\rangle|{+z}\rangle), \\ |\psi_4\rangle &= \frac{1}{\sqrt{2}}(|{+z}\rangle|{-z}\rangle - |{-z}\rangle|{+z}\rangle). \end{aligned} \tag{5}$$

By using the argument of BDSW [5], the density matrix ρ describing Alice and Bob's qubit systems can be regarded as a classical mixture of the Bell states

$$\rho = \lambda_1|\psi_1\rangle\langle\psi_1| + \lambda_2|\psi_2\rangle\langle\psi_2| + \lambda_3|\psi_3\rangle\langle\psi_3| + \lambda_4|\psi_4\rangle\langle\psi_4|, \tag{6}$$

normalized with $\sum_{i=1}^{4}\lambda_i = 1$. If we let $|\psi_1\rangle$ be the reference state, the parameters λ_1, λ_2, λ_3 and λ_4 represent the probabilities of applying the Pauli I, Z, X and Y operators to either one of the qubit of the bipartite systems. Therefore, the parameters λ_1, λ_2, λ_3, λ_4 are the probabilities of no error, only phase flip error, only bit flip error, both bit and phase flip errors, respectively. The hashing method and recurrence method have been proposed to implement the EDP in the BDSW argument [5] if the density matrix is Bell-diagonal. The job of EDP is to distill almost perfect Einstein-Podolsky-Rosen (EPR) pairs from the shared noise EPR pairs by using the local operations and classical communication to correct the bit and phase errors.

Due to the monogamy of entanglement, the eavesdropper's system almost has no quantum correlation with the system shared by Alice and Bob if they share nearly perfect pure EPR pairs. Therefore, Alice and Bob can measure the EPR pairs with the same basis to acquire the secret key while the leaked information is negligible. An important conclusion obtained in the Lo-Chau security proof [2] is that the general state (highly entangled between different pairs) brings no advantage over a mixture of products of Bell states for the eavesdropper. It successfully reduces the quantum (joint) coherent attack to classical collective attack, which means that eavesdropper's probability of cheating successfully is negligible and the extracted secret key of QKD is secure against all possible attacks by using the EDP. A drawback of the Lo-Chau security proof is the requirement of quantum computer to implement the quantum error correction (bit and phase errors). The distillation rate of EPR pairs with one-way EDP [5] in the asymptotic limit is

$$r = 1 - h(e_b) - H(e_p|e_b), \tag{7}$$

where $h(x) = -x\log_2 x - (1-x)\log_2(1-x)$ is the Shannon entropy. The conditional Shannon entropy $H(e_p|e_b)$ is given by [6]

$$\begin{aligned} H(e_p|e_b) =& -(1+a-e_b-e_p)\log_2\frac{1+a-e_b-e_p}{1-e_b} \\ & -(e_p-a)\log_2\frac{e_p-a}{1-e_b}-(e_b-a)\log_2\frac{e_b-a}{e_b}-a\log_2\frac{a}{e_b}. \end{aligned} \tag{8}$$

where $e_b = \lambda_3+\lambda_4$ is bit error rate , $e_p = \lambda_2+\lambda_4$ is phase error rate and $a = \lambda_4$ quantifies the mutual information between bit and phase errors. If the parameter $a = e_b e_p$, one has $H(e_p|e_b) = h(e_p)$, which indicates no mutual information between bit and phase errors.

The entanglement-based QKD can be reduced to prepare-and-measure protocol by exploiting the Calderbank-Shor-Steane (CSS) error correction code in the Shor-Preskill security proof [3]. One can decouple the phase error correction from the bit error correction in the CSS error correction code. Once Alice and Bob estimate the bit and phase error rates, they can choose appropriate CSS code to correct all the bit and phase errors. The phase error rate estimation method is arbitrary (direct measurement in the X basis is not necessary). The final measurement, such as the Z basis, can be moved to the beginning since the Z measurement commutes with other steps if we remove the phase error correction. Therefore, the quantum bit error correction can be replaced by classical bit error correction while the quantum phase error correction can be replaced by classical privacy amplification. For the BB84 encoding [7] with the Z and X bases, the secret key rate of the Z basis with one-way classical communication in the Shor-Preskill security proof [3] is

$$r_{\text{BB84}} = 1 - h(e_z) - h(e_x), \tag{9}$$

where $e_z = e_b$ and $e_x = e_p$ are the quantum bit error rates (QBERs) of the Z and X bases. The parameter a can be set to $e_b e_p$ in the BB84 encoding since there is no restriction on a $(0 \le a \le \min(e_b, e_p))$, which means that there is no mutual information for the worst-case scenario. The six-state [8] encoding QKD with one-way classical communication is proved by Lo [9], the corresponding secret key rate of the Z basis is

$$r_{\text{six-state}} = 1 - h(e_z) - H(e_x|e_z), \tag{10}$$

where mutual information parameter $a = (e_z+e_x-e_y)/2$ exploiting the QBER of the Y basis is $e_y = \lambda_2+\lambda_3$. One can acquire the mutual information by using the extra Y basis which means that the tolerant noise of six-state encoding is higher than the BB84 encoding.

Compared with the one-way EDP, the two-way EDP proposed by Gottesman and Lo [4] has shown an advantage in tolerating noise. Except for the final random hashing used in one-way EDP, there are another two types of steps, B step and P step, in the Gottesman-Lo security proof [4]. The B and P steps are used for decreasing the bit and phase error rates, respectively. Then the key can be extracted by applying random hashing. This is the reason why Gottesman-Lo's two-way EDP is able to tolerate more noise.

Definition of B step. Alice and Bob perform a bilateral XOR operation on two EPR pairs and compare the measurement results of target pairs in the Z basis after they randomly permute all the EPR pairs and divide them into two EPR pairs, control pairs and target pairs. The bilateral XOR measurement is used to detect the single bit error. It means that the measurement result is the same (different) given that the two EPR pairs have no bit error or both have a bit error (only one of the two EPR pairs has bit error). If the measurement outcomes are the same, they keep the control qubit; otherwise, they discard it. The B step requires two-way classical communication to change information between Alice and Bob. The B step is compatible with the prepare-and-measure protocol since the bilateral XOR operation of B step is equivalent to two measurement of $Z \otimes Z$. If we assume that the noise EPR pairs are characterized by $\{e_b, e_p, a\}$, the new state is characterized by $\{\tilde{e}_b, \tilde{e}_p, \tilde{a}\}$ [4] after one B step is applied,

$$\begin{aligned} \tilde{e}_b =& \frac{e_b^2}{(1-e_b)^2+e_b^2}, \\ \tilde{e}_p =& \frac{2(1-e_b-e_p+a)(e_p-a)+2a(e_b-a)}{(1-e_b)^2+e_b^2}, \\ \tilde{a} =& \frac{2a(e_b-a)}{(1-e_b)^2+e_b^2}, \end{aligned} \tag{11}$$

where $p_{\text{B}}^s = [(1-e_b)^2+e_b^2]/2$ is the probability of survival EPR pairs after one B step. The factor $1/2$ stems from the fact that only half of the initial EPR pairs are control pairs. For the BB84 encoding, a is a freedom parameter

$0 \le a \le \min(e_b, e_p)$, the worst case of B or P steps is $a = 0$ in the two-way EDP proved by Gottesman and Lo [4], which is different from the one-way EDP [3] with $a = e_b e_p$.

Definition of P step. Alice and Bob randomly permute all EPR pairs and divide them into three groups, one target and two control EPR pairs. They perform two bilateral XOR on three EPR pairs by one target and two control pairs. By measuring the two control pairs in the X basis and comparing the measurement results, they can find the phase error syndrome. However, the phase error cannot be detected and corrected in the prepare-and-measure protocol. The P step is reduced to implement the classical XOR operation among the three bits to generate one bit in the prepare-and-measure protocol if the Z basis measurement is performed before the P step. Therefore, if we assume that the noise EPR pairs are characterized by $\{e_b, e_p, a\}$, the new EPR pairs are characterized by $\{\tilde{e}_b, \tilde{e}_p, \tilde{a}\}$ [4] after one P step is implemented,

$$\begin{aligned}
\tilde{e}_b &= 3e_b(1-e_b)^2 + e_b^3, \\
\tilde{e}_p &= 3e_p^2(1-e_p) + e_p^3, \\
\tilde{a} &= 3a(e_p - a)(2 - 2e_b - e_p + a) + 3(e_b - a)[a^2 + (e_p - a)^2] + a^3,
\end{aligned} \tag{12}$$

where $p_{\mathrm{P}}^s = 1/3$ is the probability of survival EPR pairs after one P step since only one-third (target pairs) of the initial EPR pairs are remained.

III. SIMULATION MODEL

Similarly to the simulation of traditional QKD, we consider the case without eavesdropper's disturbance. Here, we consider that the quantum channel is a pure loss model which is similar with BS. The evolution of Fock state $|n\rangle$, coherent state $|\alpha\rangle$ and cat state after passing through the channel can be given by

$$\begin{aligned}
|n\rangle &\xrightarrow{\text{channel}} \sum_{m=0}^{n} \sqrt{C_n^m \eta_t^m (1-\eta_t)^{n-m}}\, |m\rangle_{\mathrm{T}} |n-m\rangle_{\mathrm{R}} = |\phi(n)\rangle\,, \\
|\alpha\rangle &\xrightarrow{\text{channel}} |\alpha\sqrt{\eta_t}\rangle_{\mathrm{T}} |\alpha\sqrt{1-\eta_t}\rangle_{\mathrm{R}}\,, \\
|\alpha\rangle \pm |{-\alpha}\rangle &\xrightarrow{\text{channel}} |\alpha\sqrt{\eta_t}\rangle_{\mathrm{T}} |\alpha\sqrt{1-\eta_t}\rangle_{\mathrm{R}} \pm |{-\alpha}\sqrt{\eta_t}\rangle_{\mathrm{T}} |{-\alpha}\sqrt{1-\eta_t}\rangle_{\mathrm{R}} = |\psi\rangle\,,
\end{aligned} \tag{13}$$

where C_n^m is the binomial coefficient and η_t is the transmittance of channel. The modes T and R will keep in the channel and couple to the environment, respectively. Therefore, the kept quantum states in the channel will be

$$\begin{aligned}
\rho_{\mathrm{T}}(|n\rangle) &= \mathrm{Tr}_{\mathrm{R}}\left(|\phi(n)\rangle\langle\phi(n)|\right) = \sum_{m=0}^{n} C_n^m \eta_t^m (1-\eta_t)^{n-m} |m\rangle_{\mathrm{T}}\langle m|\,, \\
\rho_{\mathrm{T}}(|\alpha\rangle) &= \mathrm{Tr}_{\mathrm{R}}\left(|\alpha\sqrt{\eta_t}\rangle_{\mathrm{T}}\langle\alpha\sqrt{\eta_t}|\,|\alpha\sqrt{1-\eta_t}\rangle_{\mathrm{R}}\langle\alpha\sqrt{1-\eta_t}|\right) = |\alpha\sqrt{\eta_t}\rangle_{\mathrm{T}}\langle\alpha\sqrt{\eta_t}|\,, \\
\rho_{\mathrm{T}}(|\alpha\rangle \pm |{-\alpha}\rangle) &= \mathrm{Tr}_{\mathrm{R}}\left(|\psi\rangle\langle\psi|\right) = |\alpha\sqrt{\eta_t}\rangle_{\mathrm{T}}\langle\alpha\sqrt{\eta_t}| + |{-\alpha}\sqrt{\eta_t}\rangle_{\mathrm{T}}\langle{-\alpha}\sqrt{\eta_t}| \pm e^{-2\mu(1-\eta_t)}(|\alpha\sqrt{\eta_t}\rangle_{\mathrm{T}}\langle{-\alpha}\sqrt{\eta_t}| + |{-\alpha}\sqrt{\eta_t}\rangle_{\mathrm{T}}\langle\alpha\sqrt{\eta_t}|),
\end{aligned} \tag{14}$$

After passing through the channel, the Fock state $|n\rangle$ will become the mixed Fock state with m $(0 \le m \le n)$ photons while the coherent state is still a coherent state containing $\mu\eta_t$ photons on average. The detection operation of threshold detector can be characterized by two measurement operators, click F^c and no click F^{nc},

$$\begin{aligned}
F^c &= \sum_{n=0}^{\infty}[1 - (1-p_d)(1-\eta_d)^n]\, |n\rangle\langle n|\,, \\
F^{nc} &= I - F^c = \sum_{n=0}^{\infty}(1-p_d)(1-\eta_d)^n\, |n\rangle\langle n|\,,
\end{aligned} \tag{15}$$

where $I = \Sigma_{n=0}^{\infty} |n\rangle\langle n|$ is the identity operator, p_d and η_d are the dark count rate and efficiency of detector, respectively.

After some calculation, the correct gain Q_Z^C and error gain Q_Z^E of the Z basis with coherent state coding can be written as

$$\begin{aligned}
Q_Z^C &= (1-p_d)[1 - (1-p_d)e^{-\mu_a\eta_a - \mu_a\eta_b}], \\
Q_Z^E &= p_d(1-p_d)e^{-\mu_a\eta_a - \mu_a\eta_b},
\end{aligned} \tag{16}$$

where $\mu_a\eta_a=\mu_b\eta_b$, $\eta_{a(b)}=\eta_d\eta_{at(bt)}$, $\eta_{at(bt)}=10^{-\beta L_{ac(bc)}/10}$, β is the the intrinsic loss coefficient of fiber channel and $L_{ac(bc)}$ is the distance between Alice and Charlie (Bob and Charlie). Similarly, the correct gain Q_X^C and error gain Q_X^E of the X basis with cat state coding can be written as

$$\begin{aligned}Q_X^C&=\frac{1-p_d}{2}\Big[1-e^{-2\mu_a-2\mu_b}-(1-2p_d)(e^{-\mu_a\eta_a-\mu_b\eta_b}-e^{-2\mu_a-2\mu_b+\mu_a\eta_a+\mu_b\eta_b})\Big],\\Q_X^E&=\frac{1-p_d}{2}\Big[1+e^{-2\mu_a-2\mu_b}-(1-2p_d)(e^{-\mu_a\eta_a-\mu_b\eta_b}+e^{-2\mu_a-2\mu_b+\mu_a\eta_a+\mu_b\eta_b})\Big].\end{aligned}\tag{17}$$

Thereby, the total gain of the Z basis, the QBERs of the Z basis E_Z and the X basis E_X can be given by

$$\begin{aligned}Q_Z&=Q_Z^C+Q_Z^E,\\E_Z&=[e_{d_Z}Q_Z^C+(1-e_{d_Z})Q_Z^E]/Q_Z,\\E_X&=Q_X^E/(Q_X^C+Q_X^E)=Q_X^E/Q_Z,\end{aligned}\tag{18}$$

where e_{d_Z} is the misalignment rate of the Z basis.

For the protocol in Ref. [10, 11] with phase-randomized coherent state $|e^{i\theta_a}\sqrt{\nu_a}\rangle_a|e^{i\theta_b}\sqrt{\nu_b}\rangle_b$, the corresponding gain can be given by

$$Q_{\nu_a\nu_b}=2(1-p_d)e^{-\frac{1}{2}(\nu_a\eta_a+\nu_b\eta_b)}I_0(\sqrt{\nu_a\eta_a\nu_b\eta_b})-2(1-p_d)^2e^{-(\nu_a\eta_a+\nu_b\eta_b)},\tag{19}$$

where $I_0(x)$ is the modified Bessel function of the first kind and $I_0(0)=1$. The density matrix of the phase-randomized coherent state is

$$\begin{aligned}\rho&=\frac{1}{4\pi^2}\int_0^{2\pi}\int_0^{2\pi}|e^{i\theta_a}\sqrt{\nu_a}\rangle\langle e^{i\theta_a}\sqrt{\nu_a}|\,|e^{i\theta_b}\sqrt{\nu_b}\rangle\langle e^{i\theta_b}\sqrt{\nu_b}|\,d\theta_a d\theta_b\\&=e^{-(\nu_a+\nu_b)}\sum_{n=0}^{\infty}\sum_{m=0}^{\infty}\frac{\nu_a^n\nu_b^m}{n!m!}|n\rangle\langle n|\,|m\rangle\langle m|,\end{aligned}\tag{20}$$

which is the mixture of Fock states. Let yield $Y_{n,m}$ denote the detection probability when Alice and Bob send Fock states with n and m photons, respectively. Therefore, the gain with intensities ν_a and ν_b can be represented by

$$Q_{\nu_a\nu_b}=e^{-(\nu_a+\nu_b)}\sum_{n=0}^{\infty}\sum_{m=0}^{\infty}\frac{\nu_a^n\nu_b^m}{n!m!}Y_{n,m}.\tag{21}$$

The yield $Y_{n,m}$ can be written as

$$\begin{aligned}Y_{n,m}=&\sum_{k=0}^{n}\sum_{l=0}^{m}\Bigg\{C_n^kC_m^l\eta_{at}^k\eta_{bt}^l(1-\eta_{at})^{n-k}(1-\eta_{bt})^{m-l}\sum_{u=0}^{k+l}\Bigg\{\frac{u!(k+l-u)!}{2^{k+l}k!l!}\left[\sum_{v=0}^{l}(-1)^{l-v}C_l^vC_k^{u-v}\right]^2\\&\times\left\{[1-(1-p_d)(1-\eta_d)^u](1-p_d)(1-\eta_d)^{k+l-u}+(1-p_d)(1-\eta_d)^u\left[1-(1-p_d)(1-\eta_d)^{k+l-u}\right]\right\}\Bigg\}\Bigg\},\end{aligned}\tag{22}$$

which can be precisely obtained by exploiting the decoy-state method [12–14] with infinite intensities. However, the tight analytical method has been provided in main text by using the three-intensity with $0<\omega<\nu$.

---

[1] B. C. Sanders, Phys. Rev. A **45**, 6811 (1992).
[2] H.-K. Lo and H. F. Chau, Science **283**, 2050 (1999).
[3] P. W. Shor and J. Preskill, Phys. Rev. Lett. **85**, 441 (2000).
[4] D. Gottesman and H.-K. Lo, IEEE Transactions on Information Theory **49**, 457 (2003).
[5] C. H. Bennett, D. P. DiVincenzo, J. A. Smolin, and W. K. Wootters, Phys. Rev. A **54**, 3824 (1996).
[6] H.-L. Yin, Y. Fu, Y. Mao, and Z.-B. Chen, Sci. Rep. **6**, 29482 (2016).
[7] C. H. Bennett and G. Brassard, in *Proceedings of the Conference on Computers, Systems and Signal Processing* (IEEE Press, New York, 1984), pp. 175–179.
[8] D. Bruß, Phys. Rev. Lett. **81**, 3018 (1998).

[9] H.-K. Lo, Quantum Inf. Comput. **1**, 81 (2001).
[10] C. Cui, Z.-Q. Yin, R. Wang, W. Chen, S. Wang, G.-C. Guo, and Z.-F. Han, Phys. Rev. Applied **11**, 034053 (2019).
[11] M. Curty, K. Azuma, and H.-K. Lo, arXiv:1807.07667 (2018).
[12] W.-Y. Hwang, Phys. Rev. Lett. **91**, 057901 (2003).
[13] X.-B. Wang, Phys. Rev. Lett. **94**, 230503 (2005).
[14] H.-K. Lo, X. Ma, and K. Chen, Phys. Rev. Lett. **94**, 230504 (2005).
